# Enhancing wheat bran deconstruction with enzyme cocktails from *Penicillium* spp. and *Trichoderma harzianum*: the impact of β-glucosidase supplementation

**DOI:** 10.1007/s42770-026-01974-0

**Published:** 2026-05-27

**Authors:** Pedro Ricardo Vieira Hamann, Hauanny Soares Martins, Paulo Henrique Cabral da Costa, Daniel Targueta de Azevedo Brito, Vinícius Rocha Cardozo da Silva, Yasmin Folha Tavares, Betania Ferraz Quirino, Eliane Ferreira Noronha

**Affiliations:** 1 Department of Cell Biology, Laboratory of Enzymology, University of Brasília, Brasília, Brazil; 2 Genetics and Biotechnology Laboratory, Embrapa-Agroenergy, Brasília, DF Brazil

**Keywords:** β-glucosidase, *Penicillium chrysogeum*, *Trichoderma harzianum*, Wheat bran, Xylanase

## Abstract

Forests and natural environments are valuable sources of organisms capable of degrading plant cell wall carbohydrates. In the present study, two isolates, *Penicillium polonicum* and *Penicillium chrysogenum*, obtained from Brazilian Cerrado soil, along with a previously characterized isolate of *Trichoderma harzianum*, were evaluated for their ability to produce carbohydrate-active enzymes when grown in the presence of wheat bran. *P. chrysogenum* exhibited higher production of endoglucanases, xylanases, and pectinases. Biochemical characterization showed that most enzymes were active at pH 5 and within a temperature range of 30–50 °C, with the exception of *P. chrysogenum* endoglucanases, which displayed optimal activity at 60 °C. Hydrolysis of cellulose and carboxymethyl cellulose by the three enzyme sources demonstrated that supplementation with recombinant β-glucosidase from *Clostridium thermocellum* (BglA) significantly enhanced reducing sugar release; specifically, when applied to *P. chrysogenum*, the reducing sugar yield from cellulose hydrolysis increased by 2.53-fold. In the hydrolysis of wheat bran, enzymes from *P. chrysogenum* and *T. harzianum* supplemented with BglA resulted in a 1.6- and 3.93-fold increase in D-glucose yield, respectively. The results presented in this study contribute to the development of more robust enzyme cocktails for wheat bran valorization.

## Highlights

• Enzymes produced by *Penicillium chrysogenum*, *Penicillium polonicum*, and *Trichoderma harzianum* exhibited biochemical properties consistent with industrial cellulase cocktails.

• Enzymes secreted by *Penicillium chrysogenum* outperformed those produced by the other fungi.

• Recombinant β-glucosidase from *Clostridium thermocellum* enhanced both total reducing sugar and D-glucose yields when applied to wheat bran hydrolysis using fungal secretomes.

## Introduction

Lignocellulosic residues from agricultural and industrial processes are among the most promising raw materials for producing value-added chemicals [1]. Currently, there is intense research focused on developing processes capable of disrupting lignocellulosic structures to release monomeric units for use in biorefineries. A major challenge in this context is the natural recalcitrance of lignocellulose, which often renders enzymatic deconstruction inefficient and economically unfeasible [2, 3].

One lignocellulosic residue that has received considerable attention is wheat bran, a coproduct of wheat processing composed largely of low commercial value fractions. This residue is abundantly available and serves as a low-cost source of dietary fiber for both human and animal nutrition [4]. Wheat bran has been investigated as a feasible lignocellulosic residue for production of enzymes [5], and biofuel generation [6], creating opportunities to add value to this by-product.

The recalcitrance of lignocellulosic biomass is mainly due to its complex composition, including polysaccharides and lignin, a heterogeneous macromolecule made up of phenolic compounds. The carbohydrate fraction of lignocellulosic biomass consists of different sugars. Cellulose, the most abundant polysaccharide, is composed exclusively of D-glucose units linked by β−1,4 glycosidic bonds [7]. Despite its uniform sugar composition, cellulose exhibits recalcitrance due to the structural organization of its fibers. These fibers contain two distinct regions: an amorphous region with a lower degree of crystallinity and a crystalline region characterized by a highly ordered structure stabilized by extensive hydrogen bonding. The crystalline region is particularly resistant to enzymatic and microbial degradation, which limits the accessibility of hydrolytic enzymes [8, 9].

The second most abundant carbohydrate component is hemicellulose, which can be composed of a linear backbone of β−1,4-D-xylose residues, known as xylan [10]. Another major class of hemicellulose is mannan, which consists of a linear backbone of β−1,4-D-mannose residues or a combination of D-mannose and D-glucose units, referred to as glucomannan [11]. Unlike cellulose, xylan often contains various side chains, including L-arabinose, D-glucuronic acid, and acetyl groups attached to its main backbone [12]. In addition to its linear backbone, mannan also contains α−1,6-linked D-galactose residues [13].

Among the lignocellulosic materials produced on a global scale, wheat bran is a widely available source of carbohydrates with significant potential for applications in biorefineries. Wheat bran accounts for approximately 14–16% of the wheat kernel and is separated from the starch-rich endosperm, making it a low-cost material [14]. Globally, it is estimated that 758 million tons of wheat are produced annually (FAOSTAT, base year 2024) [15], with wheat bran production exceeding 100 million tons per year.

In addition to its availability as biomass for biorefinery applications, wheat bran’s carbohydrate content makes it highly desirable for use as animal feed. On a weight basis, wheat bran consists of approximately 80% carbohydrates, including 5% uronic acids, 37% arabinose, 27% xylose, and 26% glucose [16]. Regarding polysaccharides, these include 19% starch, and 38% non-starch polysaccharides, which are composed of 70% arabinoxylan, 19% cellulose, and 6% β(1,3)/β(1,4) glucans. Lignin constitutes 6% [17, 18]. Additionally, its nutritional value is significant, as it contains phenolic compounds, such as ferulic acid, which may act as antioxidants [19].

A variety of enzymes are required to achieve significant plant cell wall degradation. For instance, cellulose requires enzymes that act against the amorphous regions, such as endoglucanases, as well as enzymes that target the crystalline regions, such as cellobiohydrolases. To complete cellulose hydrolysis to its monomeric unit, D-glucose, β-glucosidases must act on small cellooligosaccharides [9, 20]. In addition to classical hydrolases, cellulose can also be deconstructed by lytic polysaccharide monooxygenases, enzymes that employ oxidative mechanisms to disrupt cellulose fibers [21, 22]. Additionally, there is a group of proteins known as swollenins, which are capable of loosening cellulosic fibers and thereby facilitating the accessibility of cellulose-degrading enzymes [23]. This mechanism has been extensively studied in several organisms, including *Trichoderma reesei*, a model organism in cellulose depolymerization [24].

Although extensive research has explored organisms that degrade lignocellulose and secrete various enzymes, there is still a need for strains that can efficiently produce carbohydrate-active enzymes [25, 26]. Filamentous fungi have been the primary source of such enzymes, with *Trichoderma reesei* being a well-established and prolific secretor. In recent years, several microbiomes have been explored in the search for efficient lignocellulose-degrading organisms, including those from natural environments such as forests, soils, and the digestive tracts of animals [27, 28].

In the search for novel organisms, several fungal strains have been isolated from forest soils, offering unexplored resources for the production of plant cell wall-degrading enzymes. Numerous species have been identified, with *Penicillium* spp. frequently reported as prolific producers of hydrolases, including xylanases and endoglucanases. However, few studies have focused on isolating fungi from Brazil’s central regions (Cerrado biome), making this area a promising natural environment for discovering microorganisms with biotechnological potential. [29, 30].

Despite substantial advances in commercial cellulase cocktails in recent years, persistent bottlenecks remain [31, 32]. A major challenge is the scarcity of single microbial producers that secrete a balanced set of cellulases with sufficiently high β-glucosidase activity. β-Glucosidases catalyze the terminal conversion of cellobiose and short cello-oligosaccharides to D-glucose and, in doing so, alleviate product inhibition of other cellulase components, including cellobiohydrolases [33].

A promising strategy for creating more efficient enzyme cocktails for lignocellulose hydrolysis involves incorporating thermostable enzymes, which are not typically secreted by filamentous fungi [34]. Among the available options, enzymes produced by the thermophilic anaerobic bacterium *Clostridium thermocellum* are widely sought for heterologous expression and lignocellulose hydrolysis [8, 35]. This organism is well known for its ability to produce extracellular or cell-bound enzymatic complexes that harbor multiple glycosyl hydrolases [36, 37]. In addition to these extracellular enzymes, intracellular β-glucosidases from this organism have been extensively studied and identified as biotechnologically relevant enzymes for the final conversion of cellulose into D-glucose [38–40].

In this study, two novel isolates, *Penicillium chrysogenum* and *Penicillium polonicum*, were sourced from Cerrado soil, alongside a previously isolated strain, *Trichoderma harzianum* TR274, to produce lignocellulolytic enzymes using wheat bran as the sole carbon source. The enzymes produced were biochemically characterized and applied to the hydrolysis of lignocellulosic residues. Enzymatic mixtures were supplemented with a recombinant thermostable β-glucosidase from *Clostridium thermocellum*, and their hydrolytic efficiency was assessed. The results provide valuable insights into these novel isolates and contribute to the development of innovative enzyme blends with potential for efficient lignocellulose hydrolysis.

## Material and methods

### Isolation, identification and preservation of filamentous fungi

Filamentous fungi were isolated from Cerrado soil using MYG media, as previously described [28]. The isolates were then assessed for their ability to grow in the presence of soluble substrates, which served as indicators of carbohydrate-active enzyme secretion (results previously reported by Camargo et al. [41]). Two isolates, ISO1 and ISO16, underwent molecular identification as described in [42]. After identification, *Penicillium chrysogenum* (ISO1) and *Penicillium polonicum* (ISO16) were cryopreserved. Seven-day-old cultures of each fungus grown on MYG agar at 28 °C were washed with 0.9% (w/v) NaCl, and the resulting spores were suspended in 50% (v/v) glycerol before being stored at − 80 °C in cryovials.

### Enzyme production

For enzyme production, *Trichoderma harzianum* TR274 [43], ISO1, and ISO16 were cultivated on MYG agar plates for seven days at 28 °C [44]. After growth on solid medium, a 1 cm diameter mycelial disc was transferred to 100 mL of autoclaved minimal mineral medium containing 1% (w/v) wheat bran in a 500 mL Erlenmeyer flask [44]. Cultivation was carried out at 28 °C with agitation at 120 rpm. After seven days, the cultures were vacuum-filtered using filter paper and centrifuged at 13,000 × g for 20 min at 4 °C. The cell-free supernatant was supplemented with 0.01% (w/v) sodium azide and stored at 4 °C for enzyme characterization and hydrolysis experiments. Enzyme production was performed in biological triplicates, and the results are presented as the mean ± standard deviation of three independent experiments.

### Lignocellulosic residue handling and treatment

Wheat bran used in this study was purchased from a local market (food-grade powder) in Brasília, Brazil. It was ground using an industrial blender, autoclaved, and rinsed with distilled water until no visible impurities remained. The biomass was then dried to a constant weight and stored at room temperature until further use [45]. The biomass used in this study was not subjected to chemical pretreatment in order to preserve the full content of hemicellulose and the additional sugars from pectin.

### Enzymatic assays, and protein quantification

Hydrolytic activities against carboxymethylcellulose, birchwood xylan, and apple pectin were measured to evaluate endoglucanase, xylanase, and pectinase activities, respectively. Substrates were prepared at 1% (w/v) in 50 mM sodium acetate buffer, pH 5. Enzymatic assays were performed by adding 20 μL of enzyme solution (cell-free extract) to 60 μL of soluble substrate. Reactions were incubated for 15 min at 50 °C in a thermocycler. After incubation, 120 μL of DNS reagent was added, and the mixtures were boiled for 10 min at 100 °C [46]. The colorimetric reactions were measured in a spectrophotometer at 540 nm. Standard curves were generated using D-glucose, D-xylose, and D-galacturonic acid for the quantification of endoglucanase, xylanase, and pectinase activities, respectively. One enzymatic unit (IU/mL) was defined as the amount of enzyme required to produce 1 μmol of product per minute per milliliter of cell-free extract. All enzymatic assays were performed in triplicate. Enzymatic activity of β-glucosidase was determined using p-nitrophenyl-β-D-glucopyranoside (pNPG) as substrate, following previously described protocols [44].

Protein concentration was determined using the commercial Bradford kit (Bio-Rad, USA) following the manufacturer’s instructions [47]. A standard curve was prepared using bovine serum albumin, as described by the manufacturer. All protein quantifications were performed in triplicate.

#### SDS-PAGE

Polyacrylamide gel electrophoresis (SDS-PAGE) was performed using 12% gels under denaturing conditions in a Mini-PROTEAN system (Bio-Rad, USA) and subsequently stained with Coomassie Brilliant Blue [48].

### Enzyme characterization

To assess the influence of pH on enzymatic activity, assays for endoglucanase, xylanase, and pectinase were conducted using 100 mM sodium acetate buffer adjusted to pH values ranging from 3.0 to 8.0, similar to reported in [44]. Reactions were carried out under standard conditions at 50 °C for 15 min. The effect of temperature on enzymatic activity was evaluated in 50 mM sodium acetate buffer (pH 5.0) by varying the temperature from 30 °C to 80 °C. All assays were performed in triplicate, and the results are presented as mean values ± standard deviation.

### Cloning and heterologous expression of Clostridium thermocellum BglA

To obtain a heterologous thermotolerant β-glucosidase, the gene Cthe_0212 was amplified by polymerase chain reaction (PCR). The reaction mixture (25 µL final volume) contained: 0.4 µM of each forward (5’-AAAAACCATGGGCATGTCAAAGATAACTTTCCCA-3’) and reverse (5’- AAAAACTCGAGAAAACCGTTGTTTTTGATTACTTCT-3’) primer, 200 µM of each dNTP, 1 unit of high-fidelity DNA polymerase (Cellco, Brazil), the corresponding DNA polymerase buffer (Cellco, Brazil), 50 ng of *Clostridium thermocellum* B8 genomic DNA (previously obtained as described in [49]), and nuclease-free water to adjust the volume. PCR amplification was performed with the following program: an initial denaturation at 95 °C for 5 min; 35 cycles of denaturation at 95 °C for 30 s, annealing at 55 °C for 30 s, and extension at 72 °C for 90 s; followed by a final extension at 72 °C for 5 min. The reaction was then held at 12 °C until further use.

Following PCR amplification, the amplicon was separated on a 0.8% (w/v) agarose gel, and the corresponding DNA fragment was excised and purified using the QIAquick Gel Extraction Kit (Qiagen, USA). The purified product was digested with NcoI and XhoI restriction endonucleases for 3 h according to the manufacturer’s instructions (New England Biolabs Inc., USA). Ligation of the digested fragment into the pET-28a vector, linearized with the same restriction enzymes, was carried out using T4 DNA ligase (New England Biolabs, USA).

Screening of transformants was carried out by transforming *Escherichia coli* XL-10 Gold cells via heat shock, followed by plating on LB agar medium (0.5% (w/v) yeast extract, 1% (w/v) tryptone, 1% (w/v) NaCl, 2% (w/v) agar; pH 7.0) supplemented with kanamycin (50 µg/mL). After incubation at 37 °C for 16 h, individual colonies were transferred to liquid LB medium containing the same antibiotic and cultured at 37 °C for 16 h under agitation (200 rpm). Plasmid DNA was then extracted using the GeneJET Plasmid Miniprep Kit (Thermo Scientific, USA), and the resulting plasmid was verified by PCR using Cthe_0212 specific primers.

The construct pET-28a-Bgla was transformed into *Escherichia coli* BL21(DE3) by heat shock, and transformants were selected on LB agar medium supplemented with kanamycin (50 µg/mL). Individual colonies were inoculated into 5 mL of LB medium containing kanamycin (50 µg/mL) and cultured at 37 °C for 16 h with agitation at 200 rpm. The resulting culture was used as the pre-inoculum for heterologous BglA production. Recombinant BglA production was carried out by inoculating *E. coli* BL21(DE3)-pET-28a-BglA into 500 mL of LB medium supplemented with kanamycin (50 µg/mL). Cultures were grown at 37 °C with agitation at 200 rpm until reaching an optical density at 600 nm (OD₆₀₀) of 0.6. The temperature was then reduced to 28 °C, and protein expression was induced by the addition of lactose to a final concentration of 10 mM. After 24 h of induction, cells were harvested by centrifugation (13,000 × g, 4 °C, 20 min) and resuspended in 50 mL of 50 mM Tris–HCl buffer (pH 8.0) containing 300 mM NaCl and 5 mM imidazole, followed by cell disruption via sonication. Recombinant protein expression was evaluated by 12% SDS-PAGE followed by Coomassie Brilliant Blue staining [48].

### Heterologous protein purification

The cell lysate was centrifuged at 10,000 × g for 20 min at 4 °C. The supernatant (soluble fraction) was applied to a 5 mL HisTrap HP column (Cytiva) on an ÄKTA Purifier system. Unbound proteins were removed with 100 mL of wash buffer (50 mM Tris–HCl, pH 8.0, 300 mM NaCl, 5 mM imidazole). The bound recombinant protein was then eluted isocratically with elution buffer (50 mM Tris–HCl, pH 8.0, 300 mM NaCl, 500 mM imidazole).

Protein purity was assessed by 12% SDS-PAGE [48] and by measuring β-glucosidase activity. Fractions showing the highest purity were dialyzed against distilled water using a 10 kDa cutoff SnakeSkin™ Dialysis Tubing (Sigma-Aldrich, USA). Purified samples were stored at 4 °C until further use in hydrolysis experiments.

### Substrates hydrolysis

Initial screening of lignocellulose component hydrolysis was performed using purified soluble substrates and cellulose, namely carboxymethylcellulose, xylan, and microcrystalline cellulose. For these assays, 1 mL reaction mixtures containing 3.3 mg of each substrate, previously dissolved in distilled water, were prepared with the addition of 100 µL of each secretome (*Trichoderma harzianum*, *Penicillium polonicum*, and *Penicillium chrysogenum*) as the enzyme source. Reactions were carried out for 24 h at 50 °C in a thermomixer (1000 rpm), with the pH maintained at 5.0 using 50 mM sodium acetate buffer. Experiments were conducted in triplicate, and appropriate blanks were included for both substrates and secretomes. Reducing sugars released after hydrolysis were quantified using the DNS method [46].

A second set of experiments was conducted under the same conditions, with the secretomes supplemented with 5 IU of β-glucosidase activity from the recombinant BglA derived from *Clostridium thermocellum*. Control reactions containing only β-glucosidase were included as blanks. Results are presented as mean values ± standard deviation of the triplicate experiments.

### Lignocellulose hydrolysis

For the hydrolysis experiments, secretomes were concentrated tenfold by freeze-drying and resuspending the resulting powder in distilled water. Reactions were performed using 100 µg of protein from each secretome (*Trichoderma harzianum*, *Penicillium polonicum*, and *Penicillium chyrsogenum*) in 5 mL reaction mixtures containing 5% (w/v) wheat bran. Hydrolysis was carried out at 50 °C in 50 mM sodium acetate buffer (pH 5.0) with agitation at 150 rpm in an orbital incubator for 72 h. After the hydrolysis period, reducing sugars were quantified, and D-glucose concentrations were determined using a glucose oxidase assay (Doles, Brazil) [35]. A parallel set of experiments was conducted by supplementing the fungal secretomes with recombinant BglA from *Clostridium thermocellum*. For these assays, 10 IU of β-glucosidase activity was added to each reaction. Appropriate blanks were included, consisting of the β-glucosidase preparation alone under the same hydrolysis conditions. The β-glucosidase activity had to be doubled compared to that used in purified carbohydrate hydrolysis experiments to overcome limitations associated with lignocellulosic biomass, including unproductive interactions with plant cell wall structures.

Hydrolysis experiments were conducted in triplicate, with appropriate blanks containing only the biomass or only the secretome included as controls. Results are presented as mean values ± standard deviation of the triplicate measurements.

## Results

### Enzyme production by different fungi isolates

**Fig. 1:**
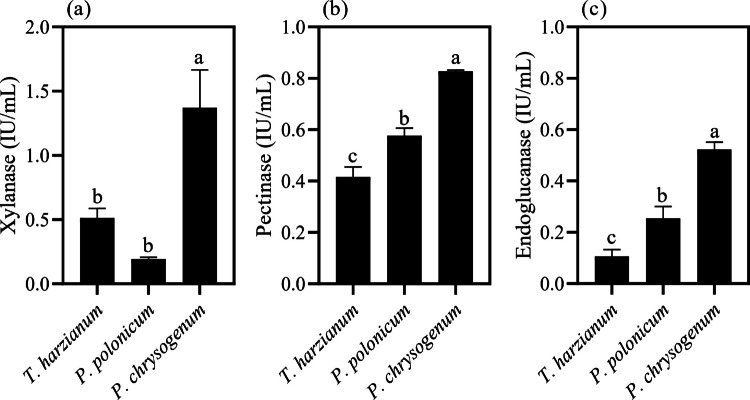
Production of plant cell wall–degrading enzymes: (**a**) xylanase, (**b**) pectinase, and (**c**) endoglucanase, by the three isolates *T. harzianum* TR274, *P. polonicum*, and *P. chrysogenum*. The isolates were cultured for seven days in liquid medium containing 1.0% (w/v) wheat bran as the sole carbon source. Standard deviations were calculated from activities measured in biological triplicates. Bars sharing the same letters within each chart indicate no significant difference (p > 0.05), according to ANOVA followed by Tukey’s test

Production of carbohydrate-active enzymes, including endoglucanases, xylanases, and pectinases, was observed for all three isolates investigated in this study: *T. harzianum*, *P. chrysogenum*, and *P. polonicum* (Fig. 1). All isolates grew in the presence of wheat bran as the sole carbon source, and enzymatic activities were detected after seven days of cultivation. Different levels of glycosyl hydrolase activities were observed, with *P. chrysogenum* showing the highest production of xylanases, pectinases and endoglucanases, reaching 1.37, 0.82, and 0.52 IU/mL (Fig. 1a, b, c), respectively. No statistically significant difference was observed in xylanase production between *P. polonicum* and *T. harzianum* (Fig. 1a). In contrast, for endoglucanase and pectinase activities, *T. harzianum* produced higher levels than *P. polonicum*, with endoglucanase activity nearly twice that produced by *P. polonicum* (Fig. 1b).

### Biochemical properties of glycosyl hydrolases secreted by filamentous fungi

**Fig. 2:**
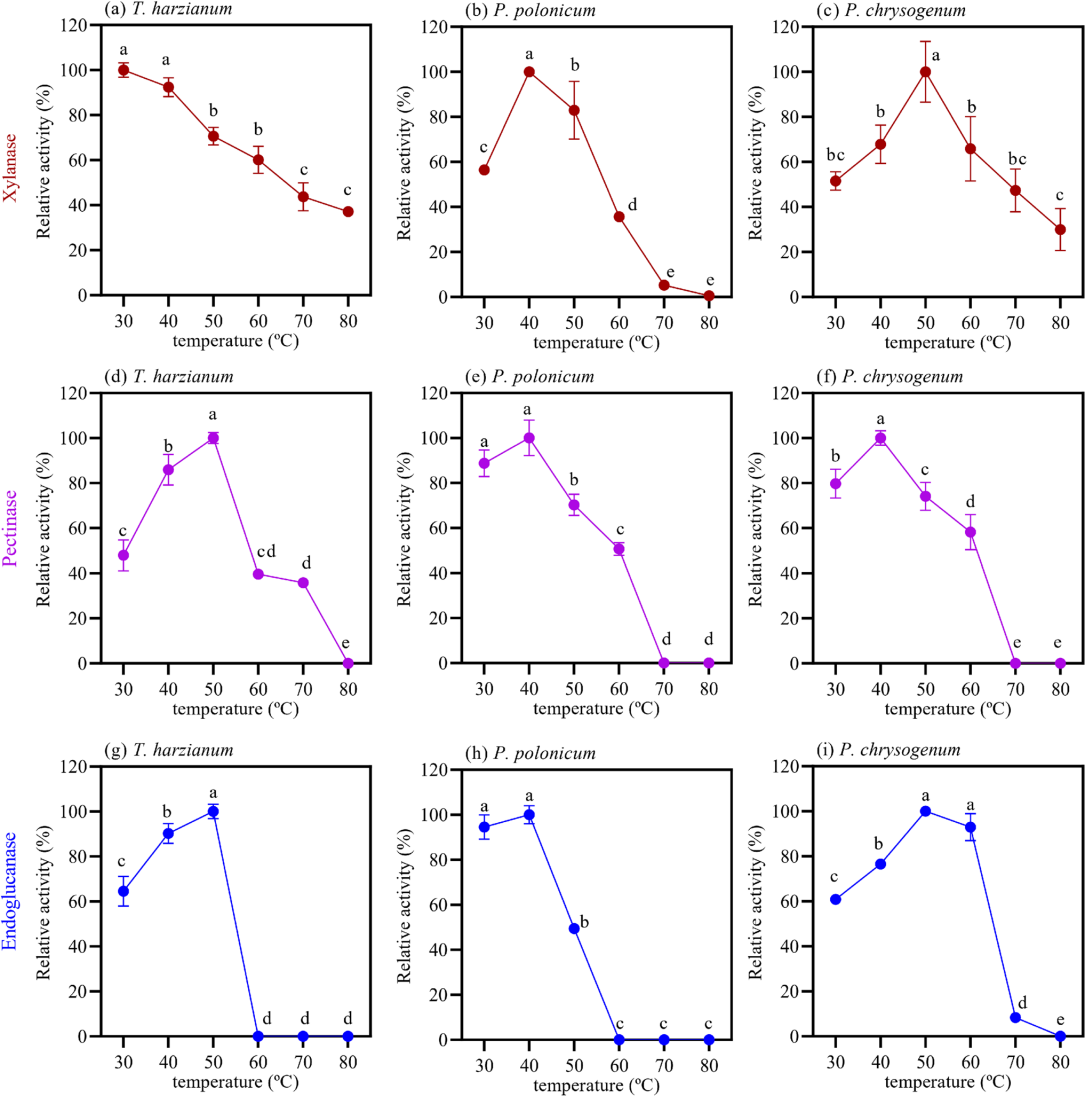
Effect of temperature on the hydrolytic activities of endoglucanase (blue markers), pectinase (purple markers), and xylanase (red markers) produced by *T. harzianum* (**a**, **d**, and **g**), *P. polonicum* (**b**, **e**, and **h**), and *P. chrysogenum* (**c**, **f**, and **i**). Enzyme assays were conducted using soluble substrates in 50 mM sodium acetate buffer at pH 5.0. Standard deviations were calculated from activities measured in technical triplicates. Bars sharing the same letter within each chart indicate no significant difference (p > 0.05) based on ANOVA followed by Tukey’s test

Endoglucanase, xylanase, and pectinase activities secreted by *T. harzianum*, *P. polonicum*, and *P. chrysogenum* during growth on wheat bran were characterized to assess the effects of temperature and pH on their hydrolytic activity. The temperature effect (Fig. 2) showed that xylanases from *T. harzianum* exhibited most of their activity within a moderate temperature range, with maxima at 30 and 40 °C (Fig. 2a). In contrast, *P. polonicum* xylanolytic activity displayed a clear optimum at 40 °C (Fig. 2b), with activities below 60% at lower temperatures and only residual activity observed at temperatures above 60 °C. *P. chrysogenum* (Fig. 2c) showed maximal xylanolytic activity at 50 °C and retained higher activity at elevated temperatures compared with *P. polonicum* xylanases.

Pectinolytic activity produced by *T. harzianum* reached its peak at 50 °C (Fig. 2d), followed by a pronounced decline at higher temperatures, retaining approximately 40% of its maximal activity at 60 °C and 70 °C. Both *P. polonicum* (Fig. 2e) and *P. chrysogenum* (Fig. 2f) exhibited similar temperature profiles for pectinase activity, with maximal activity occurring between 40 °C and 50 °C, followed by a gradual decrease up to 60 °C. No activity was detected at temperatures higher than 60 °C.

The temperature profile for endoglucanase activity showed that *P. polonicum* and *T. harzianum* secreted endoglucanases with similar performance, with most activity observed at moderate temperatures (30–40 °C) and no detectable hydrolytic activity above 60 °C (Fig. 2g and h). In contrast, *P. chrysogenum* exhibited a broader temperature range for endoglucanase activity, with peak activity between 50 and 60 °C and substantial activity still detected at 70 °C (Fig. 2i).

**Fig. 3:**
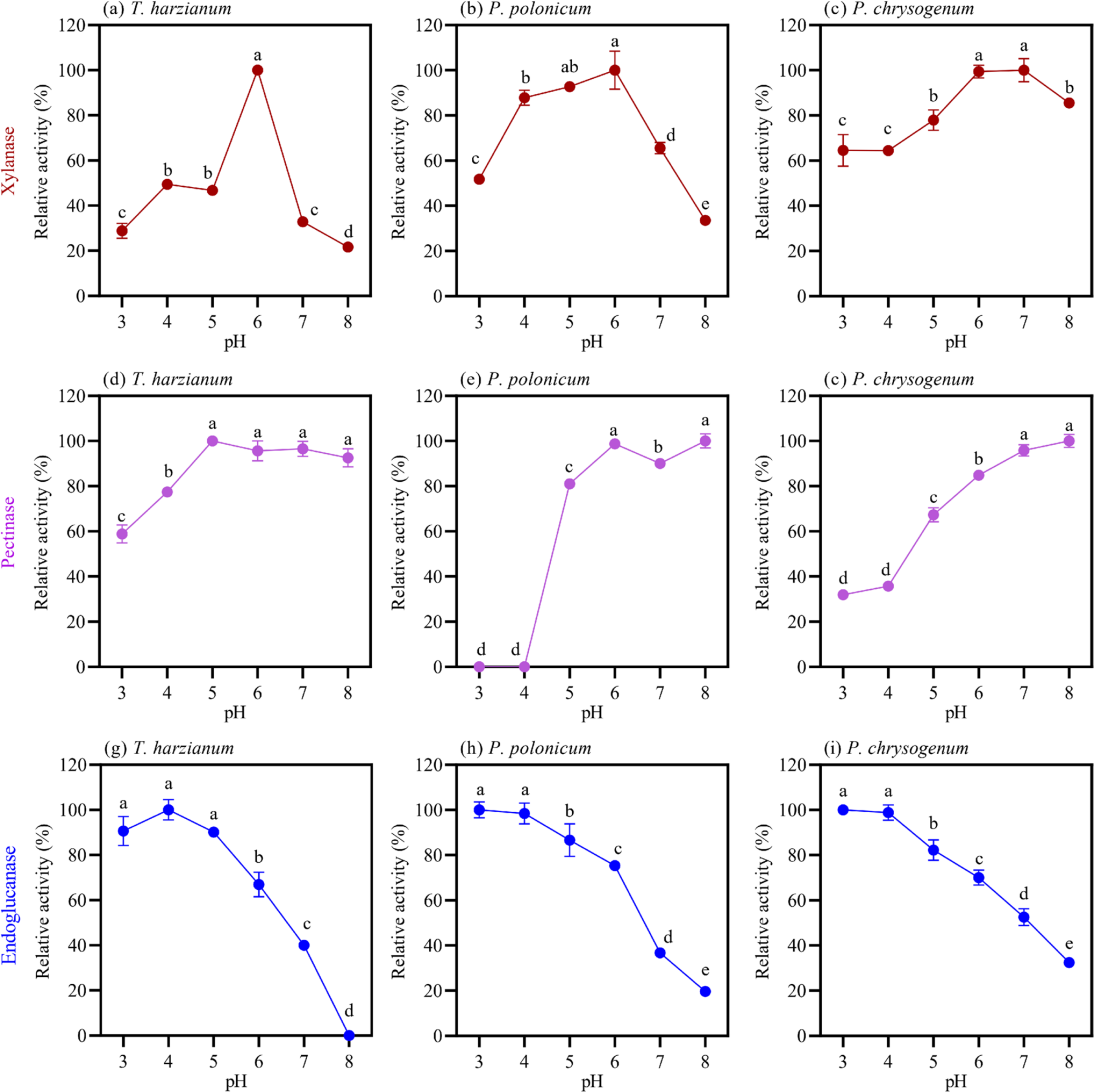
Effect of pH on the hydrolytic activities of endoglucanase (blue markers), pectinase (purple markers), and xylanase (red markers) produced by *T. harzianum* (**a**, **d**, and **g**), *P. polonicum* (**b**, **e**, and **h**), and *P. chrysogenum* (**c**, **f**, and **i**). Enzyme assays were performed using soluble substrates in 100 mM sodium-citrate buffer, with pH values ranging from 3 to 8. Standard deviations were calculated from activities measured in technical triplicates. Bars sharing the same letter within each chart indicate no significant difference (p > 0.05) based on ANOVA followed by Tukey’s test

Regarding the pH effect on carbohydrate-active enzymes secreted by the three isolates, *T. harzianum* displayed peak xylanase activity at pH 6, with approximately 50% of maximum activity observed at more acidic pH values (4 and 5) (Fig. 3a). Although peak activity occurred in the more acidic range, activity above 20% was still observed at neutral to slightly alkaline pH. *P. polonicum* exhibited xylanase activity across a broader pH range, with its maximum observed between pH 5 and 6 (Fig. 3b). In the case of *P. chrysogenum* (Fig. 3c), xylanolytic activity was more pronounced at pH values above 6, with over 80% of maximum activity achieved at pH 7 and 8.

Pectinolytic activity produced by *T. harzianum* (Fig. 3d) exhibited a broad pH range, with maximum activity between pH 5 and 8, and a noticeable decline only in more acidic conditions. A similar pH effect was observed for *P. polonicum* pectinases (Fig. 3e), with peak activity at pH 6 and 8, and no activity detected at more acidic pH values, such as pH 3 and 4. *P. chrysogenum* (Fig. 3c) exhibited a pH-dependent increase in pectinolytic activity, with activity below 40% at pH 3 and 4, and peaking in the more neutral and alkaline pH regions.

Endoglucanase activities displayed a similar pH effect across the three evaluated species, with the majority of activity observed in the acidic pH range, peaking at pH 3 to 4 (Fig. 3g, h and i). At pH 8, endoglucanases produced by *T. harzianum* were completely inactive (Fig. 3g), whereas *P. polonicum* (Fig. 3h) and *P. chrysogenum* (Fig. 3i) endoglucanases still retained activity above 20% of their maximum.

### β-glucosidase heterologous production, purification, and characterization

**Fig. 4:**
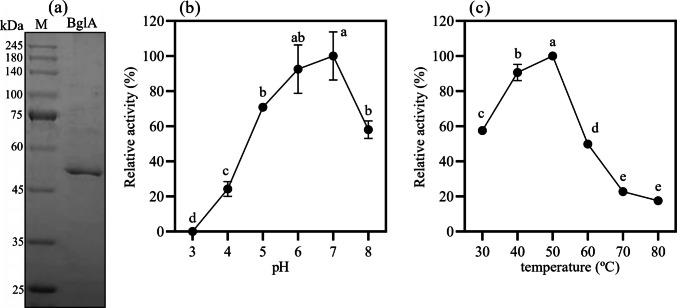
BglA purification profile on SDS-PAGE (12%) (**a**). M: protein molecular marker. Effect of pH on β-glucosidase activity of BglA (**b**) and temperature effect (**c**). Standard deviations were calculated from activities measured in technical triplicates. Bars sharing the same letter within each chart indicate no significant difference (p > 0.05), based on ANOVA followed by Tukey’s test

The recombinant protein, BglA from *Clostridium thermocellum*, was successfully produced in a soluble form, without the need for recovery from inclusion bodies. The soluble fraction exhibited β-glucosidase activity against the synthetic substrate 4-nitrophenyl-β-D-glucopyranoside and could be purified to apparent homogeneity in a single chromatography step (Fig. 4a). The molecular mass observed after SDS-PAGE was approximately 52 kDa, which corresponds to the predicted value based on the primary amino acid sequence.

Biochemical characterization of the purified recombinant protein showed that BglA has maximum activity between pH 5 and 7, with a prominent activity decay observed towards a more acidic range. Additionally, the enzyme could maintain more than 50% of its maximum activity at pH 8. Regarding the temperature effect on BglA β-glucosidase activity, maximum activity was obtained at 50 °C, and activity values below 50% were observed at temperatures of 60 °C and above. Despite the lower activity at elevated temperatures, more than 60% of maximum activity was retained at temperatures below the optimum (50 °C).

### Hydrolysis of carbohydrates and the effect of supplementation with recombinant β-glucosidase

**Fig. 5:**
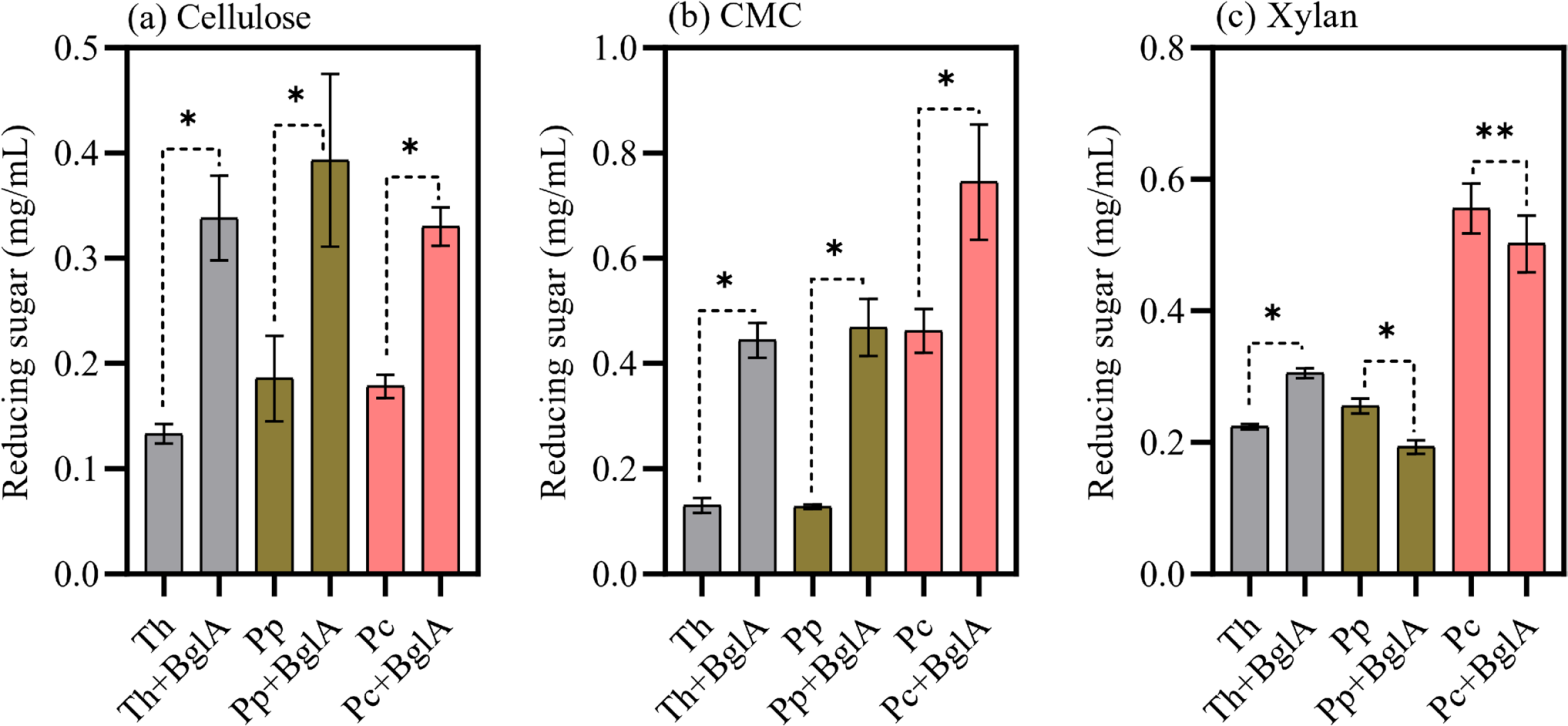
Hydrolysis yield in reducing sugars (mg/mL) from different polysaccharides: cellulose (**a**), carboxymethylcellulose (**b**), and xylan (**c**). Samples were applied as a cocktail (Th, *Trichoderma harzianum*; Pp, *Penicillium polonicum*; Pc, *Penicillium chrysogenum*) and with the addition of β-glucosidase activity (BglA). Results are presented with standard deviation from three separate hydrolysis experiments. Bars marked with * indicate significant differences (p < 0.05) after mean comparison by T-test, while ** denotes no significant difference (p > 0.05)

After enzyme characterization, the secretomes produced by the isolates were used to hydrolyze xylan, carboxymethylcellulose, and cellulose (Fig. 5). All secretomes were capable of partially hydrolyzing the evaluated substrates, with the highest hydrolysis was observed for the amorphous cellulose (carboxymethylcellulose), followed by xylan and microcrystalline cellulose.

In cellulose hydrolysis (Fig. 5a), all secretomes showed similar results in total reducing sugar release, corresponding to 0.13, 0.18, and 0.17 mg/mL, respectively, for *T. harzianum*, *P. polonicum*, and *P. chrysogenum*. Experiments with the addition of BglA resulted in a significant increase in cellulose conversion to reducing sugar, with reducing sugar levels more than twice as high as those obtained without β-glucosidase supplementation. The highest increase was observed for *T. harzianum*, which yielded 0.33 mg/mL of reducing sugar, corresponding to a 253% increase. Maximum reducing sugar with the addition of BglA was obtained from the *P. chrysogenum* secretome, with 0.39 mg/mL being produced, corresponding to a theoretical conversion yield of 10%.

Regarding carboxymethylcellulose hydrolysis (Fig. 5b), *T. harzianum* and *P. polonicum* samples showed similar release of reducing sugars, with values of 0.13 and 0.127 mg/mL, respectively. For *P. chrysogenum*, a greater amount of reducing sugar was released, reaching 0.46 mg/mL. In experiments containing the recombinant BglA, the same trend was observed. *T. harzianum* and *P. polonicum* showed almost identical improvements, reaching 0.44 and 0.46 mg/mL of reducing sugars, respectively. For *P. chrysogenum*, a significant improvement was observed, reaching 0.74 mg/mL.

Xylan hydrolysis by the *T. harzianum* and *P. polonicum* secretomes released reducing sugars at very similar yields, reaching 0.22 and 0.25 mg/mL, respectively (Fig. 5c). Upon addition of the recombinant BglA, slight differences were observed. For *T. harzianum*, a significant increase in reducing sugar release was obtained, while for *P. polonicum*, a decrease was observed. On the other hand, the enzymes from *P. chrysogenum* demonstrated excellent performance, releasing 0.55 mg/mL of reducing sugar from xylan. However, no significant difference was observed in the experiment with the addition of β-glucosidase (Fig. 5c).

### Wheat bran hydrolysis and the effect of β-glucosidase supplementation

**Fig. 6:**
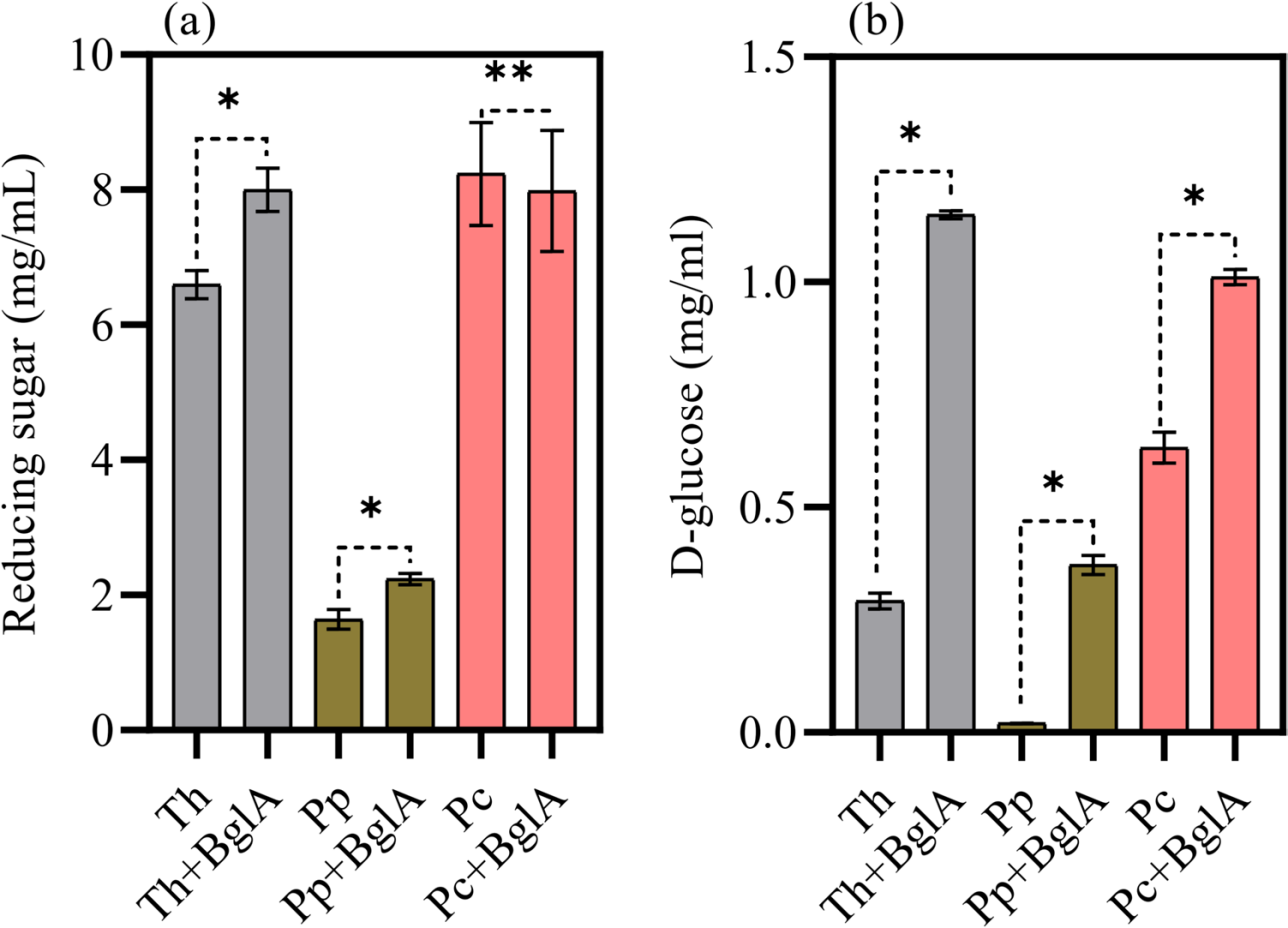
Hydrolysis yield in reducing sugars (mg/mL) (**a**) and in D-glucose (mg/mL) from wheat bran deconstruction. Samples were applied as a cocktail (*Th*, *Trichoderma harzianum*; *Pp*, *Penicillium polonicum*; *Pc*, *Penicillium chrysogenum*), and with the addition of β-glucosidase activity (BglA). Results are presented with standard deviations from three separate hydrolysis experiments. Bars marked with * indicate significant differences (p < 0.05) after mean comparison by T-test, while ** denotes no significant difference (p > 0.05)

In wheat bran hydrolysis, the enzymes from *T. harzianum* and *P. chrysogenum* exhibited the highest performance, releasing 6.59 and 8.23 mg/mL of reducing sugars, respectively (Fig. 6a). In contrast, the *P. polonicum* sample showed lower performance, releasing only 1.63 mg/mL. Regarding the effect of β-glucosidase supplementation on fungal secretomes and total reducing sugar release, no significant difference was observed for the *P. chrysogenum* sample. However, for the *T. harzianum* and *P. polonicum* samples, an increase was observed, corresponding to 7.99 and 2.23 mg/mL, respectively.

Regarding the release of D-glucose, *P. polonicum* samples produced only residual amounts of D-glucose, whereas the addition of BglA resulted in 0.37 mg/mL. For *T. harzianum*, a significant increase was observed with β-glucosidase supplementation, rising from 0.29 to 1.14 mg/mL. A similar trend was observed for the *P. chrysogenum* sample, with D-glucose yield increasing from 0.63 to 1.11 mg/mL (Fig. 6b).

## Discussion

With the growing demand for enzymes and organisms capable of efficiently producing them, various microenvironments are being explored as biotechnological sources [50]. In this study, two isolates obtained from Cerrado soil, a savanna-like biome in central Brazil, demonstrated significant potential for glycosyl hydrolase production, alongside the *Trichoderma harzianum* isolate. Cerrado soil has been investigated as a source for these organisms [41, 51], and our study shows that fungi from such environments can be harnessed for enzyme production and have substantial biotechnological potential.

The contrasting levels of glycosyl hydrolases produced by the three isolates investigated demonstrated varying aptitudes for wheat bran degradation and enzyme secretion. *P. chrysogenum* has previously been shown to be a powerful candidate for the production of glycosyl hydrolases, including xylanases and pectinases [52–54]. Our results corroborate these reports, showing that this species is a promising candidate for industrial applications as an enzyme producer.

Although *Penicillium* species are now considered effective enzyme producers as some model *Trichoderma* strains [55], *P. polonicum* exhibited the lowest enzyme production compared to *P. chrysogenum*. *P. polonicum* has been only marginally studied [56], and information on its glycosyl hydrolase production remains limited. Previous studies have demonstrated its capacity to produce xylanases [41]; however, purified enzymes alone do not fully explain the total repertoire of plant cell wall-degrading enzymes.

The most prominent activities observed for the three isolates were xylanase and pectinase, the ability of the isolates to produce greater activity targeting hemicellulose and pectin suggests a common mechanism for disrupting the less recalcitrant portions of plant cell walls, providing a carbon source for fungal growth.

The biochemical properties of the enzymes from the three isolates align well with those reported for other filamentous fungi, including the characteristics of cellulase cocktails [57, 58]. In contrast, *P. chrysogenum* exhibited cellulases with activity at higher temperatures, a feature rarely observed in *Penicillium* species, with only one report of such activity in *Penicillium funiculosum* [59]. The prevalence of xylanase activity at mild temperatures in *T. harzianum* points out the potential of these enzymes for applications beyond general degradation for biofuel production. Specifically, they could be further studied for supplementation in ruminant feed, where enzymes with optimal activity between 30 and 40 °C are highly desired [60, 61]. Additionally, the xylanase activity at temperatures as low as 30 °C makes the *Trichoderma harzianum* secretome an attractive candidate for hydrolyzing xylan, further enabling the use of these sugars for fermentation by engineered yeasts [62].

Recombinant production of BglA from *Clostridium thermocellum* resulted in a protein with high homogeneity, demonstrating the feasibility of this process and facilitating the production of recombinant β-glucosidases with industrial relevance. Initial reports of β-glucosidases produced by *C. thermocellum* involved intracellular production by the native bacteria, which resulted in low protein yields and hindered scalability to an industrial level [39]. In this regard, the heterologous production of such proteins enables their production at an industrial scale. The biochemical properties observed for the recombinant BglA align with those of other recombinant β-glucosidases from *C. thermocellum* [40]. The pH and temperature profiles are consistent with those of other microbial β-glucosidases [63], and the range at which maximum activity is achieved corresponds to the general biochemical properties of commercial cellulase cocktails. This makes the enzyme a strong candidate for incorporation into cellulosic enzyme mixtures.

Hydrolysis of carbohydrates revealed that all evaluated cocktails were deficient in β-glucosidase activity, and supplementation of this activity is crucial for the deconstruction of both amorphous and microcrystalline cellulose. The lower percentage increase in cellulose hydrolysis observed in *P. chrysogenum* enzymes when supplemented with β-glucosidase, compared to the other isolates, may be a direct consequence of its natural production of β-glucosidase, as previously reported. [44]. The presence of native β-glucosidase may enhance the final yield by alleviating the inhibition on cellobiohydrolases. Therefore, the addition of external β-glucosidases may result in a less pronounced boosting effect.

Although β-glucosidase supplementation improved the overall yield of cellulose-based component hydrolysis, the similar yields observed for all three samples in experiments without supplementation suggest that other enzymes, in addition to β-glucosidase, may be absent in all three samples. This includes a potential deficiency in lytic polysaccharide monooxygenases (LPMOs), which could limit further increases in the complete hydrolysis of cellulose.

The significant difference observed in xylan hydrolysis when using enzymes secreted by *P. chrysogenum* compared to the other strains further reinforces the hemicellulolytic potential of this species. This enhanced ability to deconstruct xylan-based hemicellulose may be a direct consequence of the variety of accessory enzymes produced by *P. chrysogenum*. For instance, this species has been extensively investigated for its production of feruloyl esterase, a key enzyme in xylan debranching [64].

Hydrolysis of wheat bran revealed the superior performance of *P. chrysogenum* and *T. harzianum* enzymes over those of *P. polonicum*. A significant difference from *P. polonicum* was not anticipated, as this species showed better enzyme production performance compared to *T. harzianum*. This effect may be attributed to the presence of distinct sets of cellulases and accessory enzymes involved in xylan deconstruction, as previously reported for *P. chrysogenum*. The complexity of wheat bran as a raw material for enzymatic hydrolysis was also expected, given that no chemical pretreatment was applied to the biomass. Nevertheless, both *P. chrysogenum* and *T. harzianum* enzymes were able to generate significant amounts of reducing sugars.

The D-glucose yield observed for *T. harzianum* and *P. polonicum* enzymes during wheat bran hydrolysis indicates that these isolates can natively secrete enzymes capable of generating D-glucose. However, the inability of *P. polonicum* to produce D-glucose in the absence of external β-glucosidase supplementation suggests that this isolate may not be the most suitable candidate for the production of enzyme systems aimed at complete lignocellulosic deconstruction. Nevertheless, enzyme cocktails lacking β-glucosidase activity may still be effectively applied in other processes, such as probiotic formulation and the development of animal feed additives [60, 61].

β-glucosidase incorporation into enzyme cocktails produced by fungi has been reported for several isolates, such as *Trichoderma reesei*, a species known for its high cellulase production but low β-glucosidase activity [65]. However, most of these reports focus on fungal β-glucosidases, and the increase in D-glucose yield observed in some cases is lower than that observed in the present study [66]. The higher increment in D-glucose yield observed with the addition of BglA may result from a combination of factors, including the generally lower β-glucosidase activity seen in filamentous fungi. Additionally, since BglA is derived from a thermophile, its heat resistance allows the enzyme to function alongside fungal cellulases for a longer period, generating more D-glucose and alleviating cellobiose inhibition on cellobiohydrolases.

## Conclusion

The three isolates evaluated in this study were capable of producing a broad array of carbohydrate-active enzymes with activity against various plant polysaccharides. The biochemical properties of these enzymes were generally consistent with those reported for commercial cellulase cocktails, with the exception of *Penicillium chrysogenum* cellulases, which exhibited activity at elevated temperatures. Supplementation with recombinant β-glucosidase demonstrated that all enzyme cocktails require this activity to enhance the hydrolysis of cellulose and carboxymethyl cellulose. Although all isolates were capable of hydrolyzing wheat bran, *P. polonicum* was unable to release D-glucose. This finding highlights the potential application of these enzyme cocktails in other processes, such as the production of animal feed additives. Future studies will focus on the incorporation of additional recombinant activities, such as cellobiohydrolases, to complement β-glucosidases and further enhance wheat bran hydrolysis. Additionally, enzyme production will be conducted in bioreactors to scale up production and evaluate hydrolysis in industrial settings.

## Data Availability

The authors affirm that all data generated and analyzed during the course of this study are fully contained within the manuscript.
